# Delivery of stable ultra-thin liquid sheets in vacuum for biochemical spectroscopy

**DOI:** 10.3389/fmolb.2022.1044610

**Published:** 2022-11-14

**Authors:** Jonathan C. T. Barnard, Jacob P. Lee, Oliver Alexander, Sebastian Jarosch, Douglas Garratt, Rose Picciuto, Katarzyna Kowalczyk, Clement Ferchaud, Andrew Gregory, Mary Matthews, Jon P. Marangos

**Affiliations:** Extreme Light Consortium, Blackett Laboratory, Imperial College London, Department of Physics, London, United Kingdom

**Keywords:** XUV spectroscopy, spectroscopy in liquids, microfluidics, liquids in vacuum, interferometry, liquid flatjet

## Abstract

The development of ultra-thin flat liquid sheets capable of running in vacuum has provided an exciting new target for X-ray absorption spectroscopy in the liquid and solution phases. Several methods have become available for delivering in-vacuum sheet jets using different nozzle designs. We compare the sheets produced by two different types of nozzle; a commercially available borosillicate glass chip using microfluidic channels to deliver colliding jets, and an in-house fabricated fan spray nozzle which compresses the liquid on an axis out of a slit to achieve collision conditions. We find in our tests that both nozzles are suitable for use in X-ray absorption spectroscopy with the fan spray nozzle producing thicker but more stable jets than the commercial nozzle. We also provide practical details of how to run these nozzles in vacuum.

## 1 Introduction

With the proliferation of lab-scale high harmonic generation (HHG) sources capable of producing isolated sub-femtosecond X-ray pulses (Goulielmakis et al., 2008) and the recent advent of X-ray free electron laser (XFEL) technology capable of producing the same (Joseph et al., 2020), ultrafast X-ray spectroscopy is able to provide unparalleled access to the electronic states and dynamics of a system during photon initiated reactions with sub-femtosecond resolution. Recent work has elucidated ultrafast exciton localisation in organic semiconductors (and other solid examples) (Garratt et al., 2022), and many experiments have been performed in the gas phase following ionisation or excitation and tracked the subsequent electronic and structural changes (Attar et al., 2015; Schnorr et al., 2019).

There are important motivations for using liquid targets for X-ray experiments, given the access to element-specific and ultrafast dynamics these experiments can provide. The majority of important and under-studied chemical and biological processes occur in the solution phase, so in order to provide results that are more representative of real systems, a solution-phase target is desirable. The presence of neighbouring molecules, either solvent or other solutes, is known to have a large effect on electron dynamics compared to isolated systems (Jordan et al., 2020). As liquid targets refresh themselves between shots they are self-healing, so the concerns about damage or photobleaching that exist in solid targets are avoided with flowing liquid targets, allowing for experiments with stronger fields.

Access to photo-induced dynamics in the liquid or solution phase at few-femtosecond timescales *via* soft X-ray absorption spectroscopy is still in its infancy, with the first reported study in 2020 (Smith et al., 1988). This is partly due to the difficulty of delivering thin liquid targets in vacuum, where extreme ultraviolet (XUV) and soft X-ray beams can be used. If using an HHG source X-ray flux becomes a key concern, so the absorption due to silicon nitrate windows in flow cells or the relatively thick conventional cylindrical jets can render signal transmission close to zero, and furthermore the curvature of the surface in the cylindrical jets induces a focusing that can lead to beam distortion. Spherical droplets suffer the same drawback, significant focusing that ionises the droplet, and scattering of the detected light. Therefore, for soft X-ray absorption spectroscopy with HHG sources windowless, stable and thin flat sheets (or flatjets) are preferable.

Several liquid sheet jet designs capable of producing sheets on the order of 1 µm have been published (Ekimova et al., 2015; Galinis et al., 2017; Ha et al., 2018; Koralek et al., 2018). This paper discusses the working principles and design of two types of microfluidic jets, comparing the dimensions and stability of the sheets produced, in particular focusing on vacuum performance essential for XUV spectroscopy. These types are a gas dynamic virtual nozzle (GDVN) first presented by Koralek et al. (2018), and a fan spray type nozzle first presented by Galinis et al. (2017). In both jets the liquid sheet forms on the exit to the nozzles and this work therefore complements a recent study by Chang et al. (2022), where the temperature dependence of sheets produced by both the externally colliding jets and the GDVN was examined. We also present details of our vacuum system and some pointers to address common pitfalls of using these targets in vacuum.

## 2 Materials and methods

**FIGURE 1:**
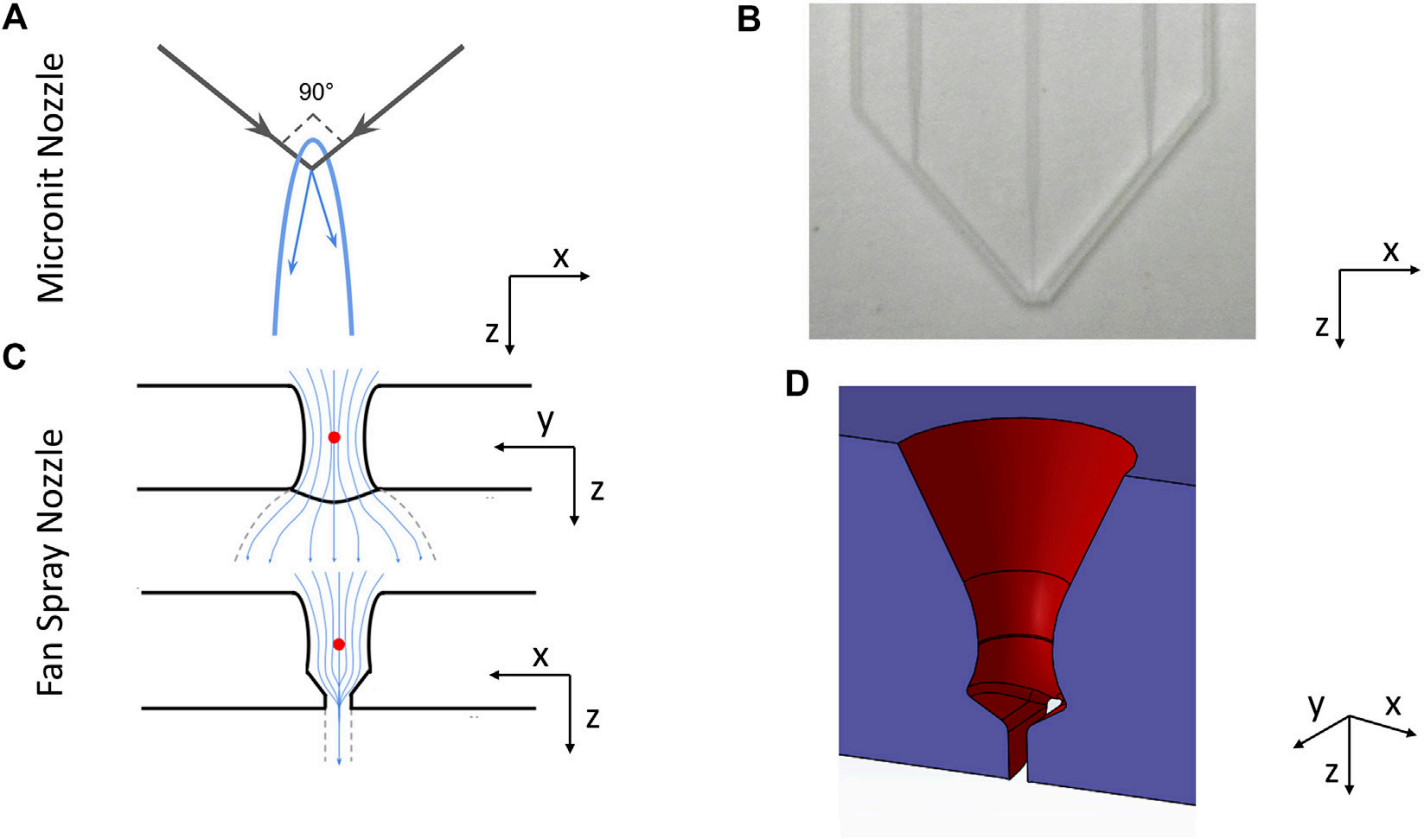
**(A)** shows a schematic illustration of how colliding jet sheet jets work—the liquid collides at an angle, spreading out in a sheet in the plane of collision. **(B)** shows a picture of the Micronit nozzle. The central channel is intended for liquid, whilst the two outside channels are intended for gas. We normally operated it by leaving the central channel empty and flowing liquid through the two outside channels. **(C)** shows cross sectional slices of the fan spray nozzle, showing the path taken by the liquid inside the nozzle in the x- and y-planes. **(D)** shows a cutaway of the CAD drawing of the fan spray nozzle, showing the cone narrowing and spreading out again, and the stabilising wall at the nozzle exit.

The basic principle for creating a liquid flatjet relies on appropriate momentum transfer to cause thinning and flattening in one direction. Two streams of liquid collide at a controlled angle. The opposing horizontal momentum components cancel out, which leads to the sheet spreading out radially in the plane of the collision due to the incompressability of the liquid. If the angle between the flows of liquid that collide is not 180° (i.e., there is a pre-existing momentum component in the plane of collision), the jet will predominantly spread out in the direction of this momentum component (see Figure 1A). As the sheet spreads out, its thickness, t, is inversely proportional to the distance from the point of the collision as the amount of liquid in the sheet is conserved. Surface tension acts to oppose the spreading, providing the force which draws the sheet back to a point, resulting in the formation of an acute leaf shape and limiting how thin the sheet can be. Hence, the ultimate thickness of the sheet depends on the volume of liquid flowing through the collision point. In order to make the sheet as thin as possible, it needs to be produced with a small volume of liquid, which necessitates the nozzle dimensions being very small, of the order of tens of µm.

Flatjet sheets employed in X-ray spectroscopy and other measurements must also be highly stable. Here, stability is defined by the sheet holding constant thickness and shape for the duration of a measurement, being resistant to break-up and phase changes, as well as maintaining a flat, ripple-free surface with respect to the wavelength and beam size. The primary breakup mechanism that we have observed for these jets arises due to asymmetric surface waves. If these surface waves become large enough relative to the sheet thickness it will cause the sheet to break up into droplets (Huang, 1970; Eggers and Villermaux, 2008), but also they can fluctuate the absorption path length leading to increased noise in absorption measurments. A measure of the relationship between the inertia of the liquid and the viscosity of the liquid is given by the Reynolds number:

$$\text{Re}=\frac{\rho uL}{\mu },$$ (1)

where *ρ* is the density of the liquid, *u* is the velocity of the liquid, *L* is a characteristic length defined by the parameters of the system under study and *μ* is the dynamic viscosity of the liquid. Although there are a number of factors that could influence the exact location of the threshold, an estimated figure for the point at which surface waves will cause breakup is given by Huang (1970) as a Reynolds number of approximately 2,000, which is close to the value obtained from our results presented later on in this paper.

### 2.1 Methods to generate flat sheet jets

The nozzle designs both operate according to the same principles outlined above. However they differ in their details, most notably in their collision mechanisms. The Micronit chip nozzles consist of two plates of borosillicate glass with 50 µm wide channels etched into them. Two channels are set at 90° to each other, whilst the third channel is positioned in between these at 45° to both channels, Figure 1B. This design was first presented by Koralek et al. (2018), and nozzles are commercially available from Micronit GmbH.

The original intended working mode of this nozzle is to flow liquid down the central channel and gas down the side channels. The momentum of the gas imparts the radial momentum to the liquid, causing it to spread out into the thin flatjet. However in practice the high gas pressures required add complexity and the sheets produced are very small. Thus, we have run this nozzle by flowing liquid through the gas channels which is closer to a “true” collision regime. The momentum that creates the sheet is provided by the streams of liquid themselves, and this method works reliably to produce sheets of desired thickness and stability. In a subsequent paper the authors have improved on this design (Crissman et al., 2022), however at this time the improved design is not available commercially.

The second nozzle was created in-house, as presented in Galinis et al. (2017). This nozzle works by funnelling liquid down into a compression point *via* a cone with an angle 60° to the normal. As shown in Figure 1C, the liquid spreads out uninterrupted along the *y*-axis from this point, allowing the liquid to create a sheet. In the *x*-axis however, the liquid starts to spread out before cutting sharply back in, cancelling out the transverse momentum and ensuring that the sheet spreads out as thin as possible. This design is similar to nozzles used to spray droplets for fuel injection or pesticide spreading (Dombrowski et al., 1960), so it is referred to as a “*fan spray*” nozzle. A cutaway of this nozzle design is shown in Figure 1D. This specific nozzle is not available commercially, but similar can be manufactured using a commercially available 3D printer (Photonic Professional GT2, by Nanoscribe GmbH, or *via* ProForNano).

## 3 Results

### 3.1 Characteristics of the sheets produced by the microfluidic apparatus

**FIGURE 2:**
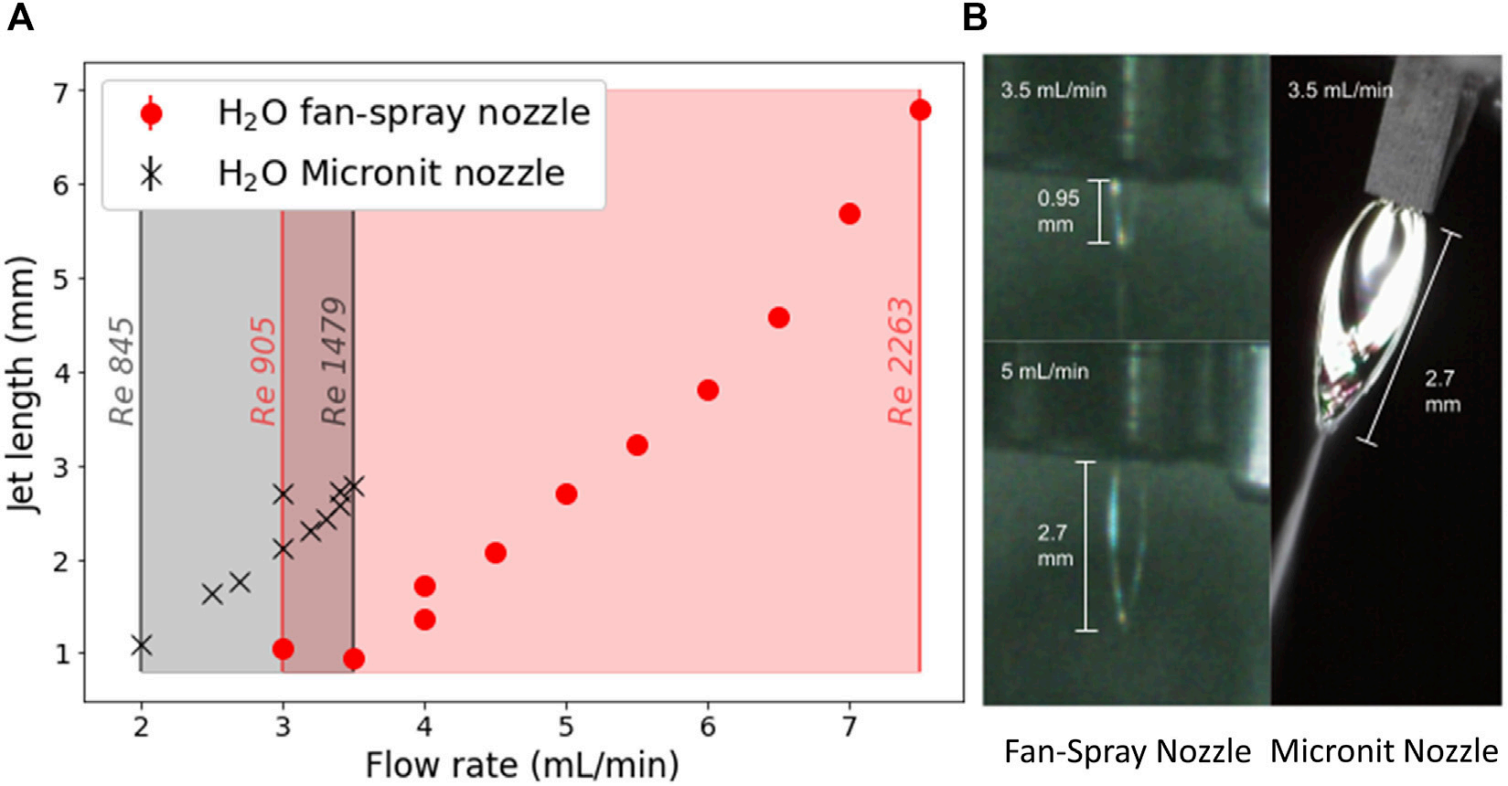
**(A)** A graph of the length of the water jet against flow rate for the Micronit nozzle (black) and the fan spray nozzle (red). The length of the jet was measured from the images using ImageJ and scaled using reference objects in the same image. The flow rate was measured at the HPLC pump head, and was increased until the sheet became unstable. The Reynolds numbers shown on the graph were calculated using a characteristic length of 50 μm and 70 µm for the Micronit and fan spray nozzles respectively, and 20°C water. **(B)** Images showing the length of a sheet produced by the Micronit nozzle at 3.5 ml/min (right), and the fan spray sheet for the same flow rate (top left) and with the same length (bottom left). The Micronit nozzle sheet has noticeably more structure and is clearly wider than the sheets produced by the fan spray nozzle.

Due to the differences in geometry, the sheets produced by the two nozzles have somewhat different properties. Figure 2 shows a comparison of the length of the sheets produced using water by these two nozzles. The most obvious difference is in the size of the sheets produced—the sheets produced by the Micronit nozzle are significantly longer than those produced by the fan spray nozzle for flow rates below 3.5 ml/min. Beyond this flow rate the sheet produced by the Micronit nozzle becomes unstable. This occurs at 7.5 ml/min for the sheet produced by the fan spray nozzle. Surface waves are noticeably more prominent on the sheets produced by the Micronit nozzle than on those produced by the fan spray nozzle, which is the probable source of instability.

These differences in jet structure can be ascribed to the differences in the geometry around the collision point in the two nozzles. Although the Micronit nozzle does guide the sheet after collision in some sense, since the liquid is prevented from spreading out in all directions freely due to the presence of the chip itself, it is not designed to actively shape the sheet. Thus, the liquid spreads out approximately as a sheet produced by the standard colliding jets geometry (Taylor, 1959a; Taylor, 1959b; Taylor, 1959c; Taylor, 1960). The fan spray nozzle, however, does purposefully guide the sheet into a desired shape. As shown in Figure 1, after the collision point, the nozzle opens up again at the same angle. It then cuts back to form the slit in one of planes which is effectively a 50 µm long wall on either side of the sheet. This has the effect of determining the maximum thickness of the sheet as it starts to spread out, and also damps any surface waves that may form due to the momentum transfer inside the nozzle.

The greater length of the sheets from the Micronit nozzle at a constant flow rate can be explained as a function of the smaller diameter of the channels that liquid flows through in the chip. They are slightly elliptical, with a major axis of 50 µm and a minor axis of 45 μm, whilst the fan spray nozzle has a minimum diameter of 70 µm. This smaller diameter means that in order to maintain the same flow rate, the liquid needs to flow at a greater velocity. Therefore, at the point of collision there is a greater amount of transverse momentum to be distributed, leading to a larger sheet, and therefore a thinner sheet. As noted in Figure 2, a Reynolds number of around 1,500 the Micronit nozzle sheet becomes unstable, whereas the fan spray sheet is stable up to around 2,300. This extended stability into higher Reynolds numbers can be attributed to the damping effect of the walls in the nozzle damping out the surface waves.

**FIGURE 3:**
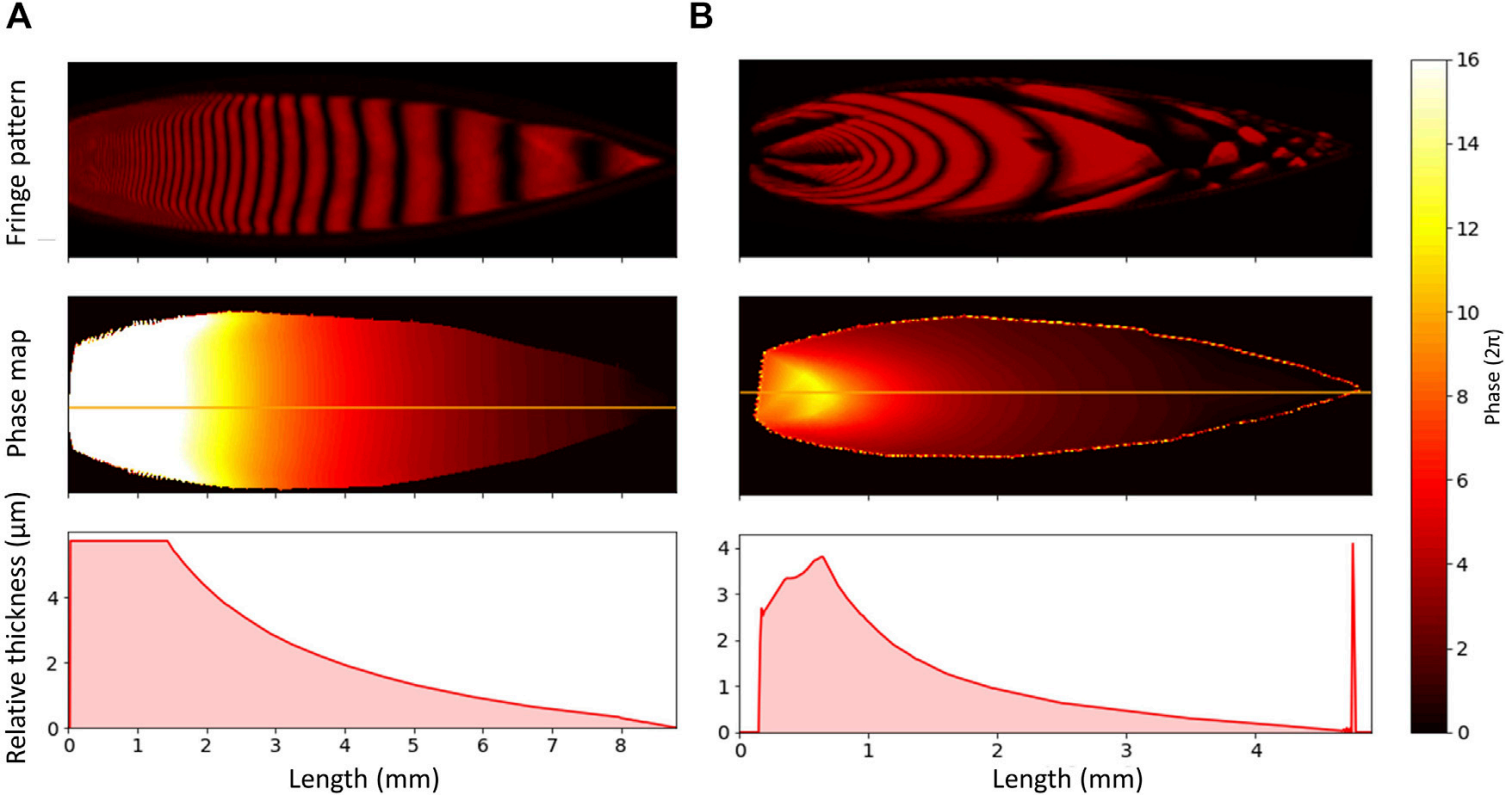
Monochromatic interferograms produced by illuminating isopropanol with a 633 nm HeNe laser. **(A)** uses the fan spray nozzle, and **(B)** uses the Micronit nozzle. Below the fringe patterns are some phase maps, produced from the fringe patterns using Jakub Dranczewski’s Magic2 package (Dranczewski, 2021). The bottom panel shows the change in relative thickness along the centerline of the sheet, indicated on the phase map. The phase near the nozzle becomes unreliable due to the fact that the lines become so close together that the camera cannot resolve them properly. In reality, the thickness should continue to increase smoothly. The spike in the thickness profile in column **(B)** is an artefact due to light scatter from the rim of the sheet.

To compare the sheet thickness we performed interferometry measurements (Galinis et al., 2017) for sheets produced in isopropanol, with the results shown in Figure 3. The sheet produced by the fan spray nozzle has a shallower gradient along its length than that produced by the Micronit nozzle. The guidance provided in the fan spray nozzle restricts the direction that the liquid can flow, and so we can assume that a greater proportion of the liquid just after the nozzle exit is in the sheet rather than the rims compared to the Micronit nozzle. The liquid then migrates to the rims more gradually, leading to this shallower decay curve.

### 3.2 Operation of microfluidic jets in vacuum

In order to produce a sheet from these nozzles, pressurised liquid needs to be forced through them. This can be achieved using compressed gas, or using an HPLC (High Pressure Liquid Chromatography) pump (Knauer Azura 6.1 L). We preferred the HPLC pump as it allows for greater control over the flow rates and access to higher backing pressures. The pulsation due to the HPLC pump is damped using a corrugated pipe and an analog pressure meter (Ventura and Nikelly, 1978) inserted between the pump output and chamber input. Blocking of the nozzles is minimised by two 2 µm pore inline filters in the high pressure line before the nozzle.

**FIGURE 4:**
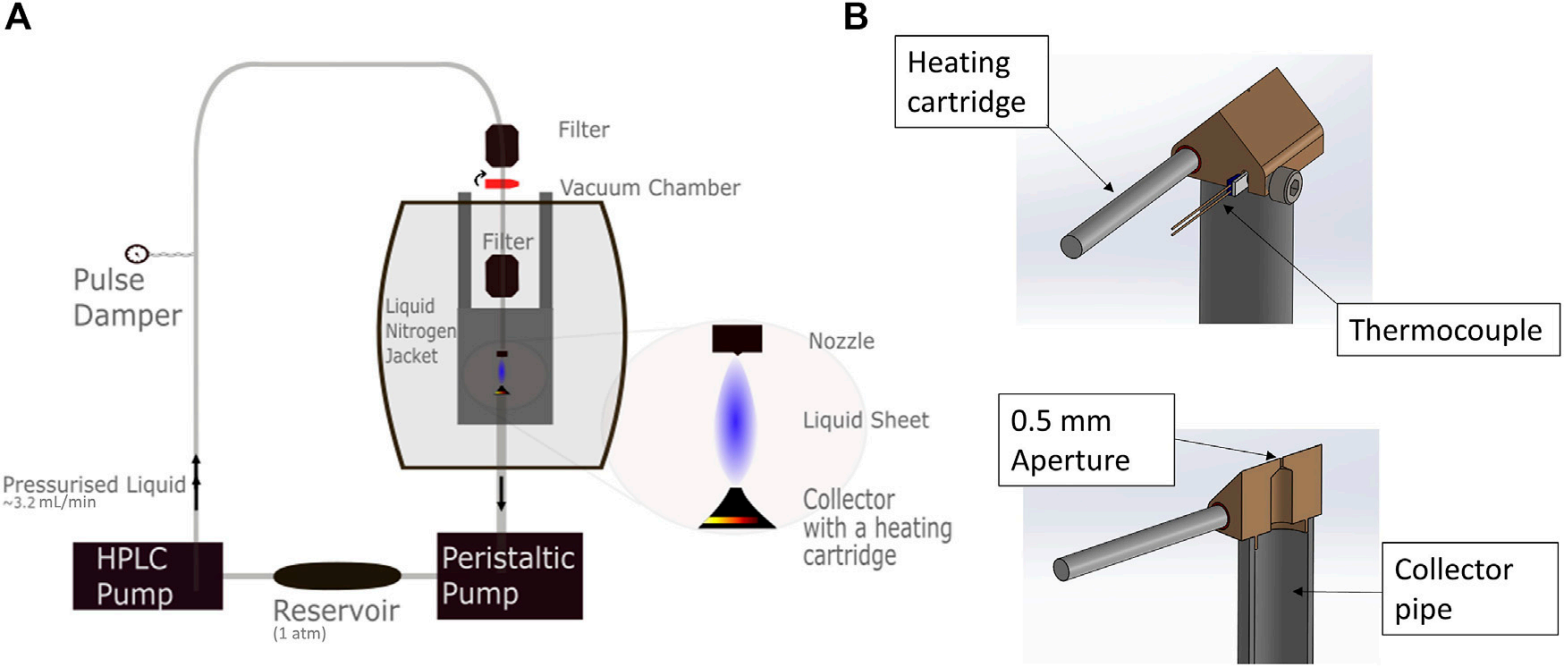
**(A)** A sketch of our recirculating system. The liquid is pumped from an atmospheric pressure reservoir by the HPLC pump into the nozzle, where it creates the sheet. The collector tip is heated to 60° by a 25 W heating cartridge. A thermocouple is attached to the collector tip to ensure a constant temperature is maintained. The liquid is then pumped from vacuum to atmosphere by a peristaltic pump (Gilson Minipuls 3). The analogue pulse damper is attached to the high pressure side, and eliminates the pulsation due to the HPLC pump. **(B)** Schematics of our catcher head, showing the heating cartridge and thermocouple attachments for temperature stabilisation, and the small aperture to reduce backflow.

Our recirculating system is shown in Figure 4. The liquid is pumped from the atmospheric pressure of the reservoir through the nozzle, where it produces a sheet. This sheet is then fed into the catcher, taking care that the tip of the leaf is located in the aperture to minimise splashing. The catcher is a triangular prism made of phosphor bronze (for greater thermal conductivity). The aperture is small (0.5 mm diameter) to prevent gas backflow from the pipe below the catcher, and the catcher also has a resistive heating tube and a thermocouple installed in order to control its temperature. The liquid caught by the catcher is then pumped back to the atmospheric pressure reservoir by a peristaltic pump (Gilson Minipuls 3), which allows for recirculation.

However, recirculation of volatile liquids, which includes many common solvents, still provides a significant challenge. Due to the geometry of our chamber the distance between the collector and the peristaltic pump is around a meter, which is a great enough distance for a high vapour pressure liquid to evaporate before it is pumped into the reservoir. Recirculating these liquids requires attention to the rate of evaporation, making the distance from the collector to the peristaltic pump as short as possible and optionally cooling the collector to further reduce the volatility.

The vacuum in the chamber is maintained by a liquid nitrogen jacket, which surrounds the liquid jet apparatus and traps most of the vapour. In this way we are able to maintain the ambient pressure in the chamber at 1 × 10^−3^ mbar or below.

### 3.3 X-ray absorption in ethanol

**FIGURE 5:**
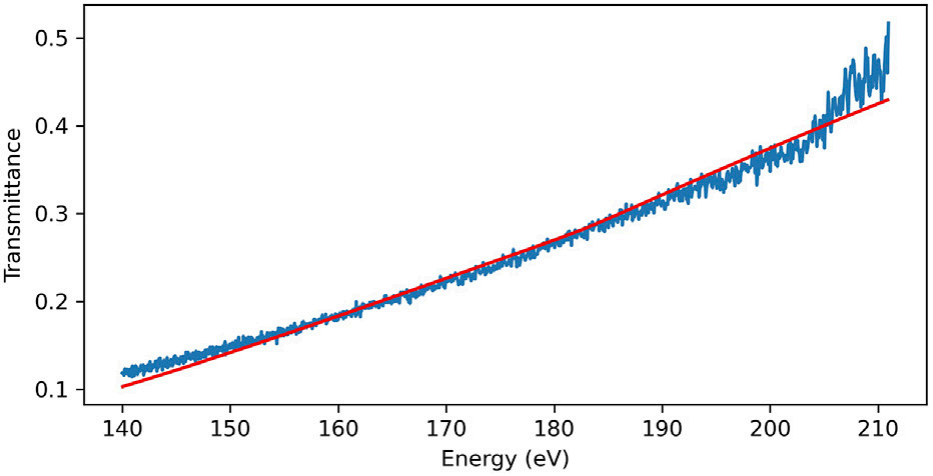
An X-ray transmission spectrum taken using the fan-spray jet and our soft X-ray beamline (Johnson et al., 2018). The red line is the calculated absorption spectrum for a 1.6 µm thick slab of ethanol using data from Henke et al. (1993). The discrepancies below 150 eV and above 200 eV are due to a lack of signal in those energy regions owing to absorption in the Zr filter we used to get rid of the driving laser field.

The purpose of these flatjets is to perform soft X-ray spectroscopy on them, and an X-ray transmission spectrum of ethanol is shown in Figure 5. The ethanol sheet was created using the fan-spray nozzle and this spectrum was taken using soft X-rays produced by HHG in neon. The jet was aligned such that the X-rays passed through the center of the sheet, and the measured transmission coefficients agree well with calculated ones based on data from (Henke et al., 1993) for a 1.6 µm thick sheet. This value accords well with the thickness values measured by interferometry in (Galinis et al., 2017), and as such provides us with an independent verification of the thickness of our jet in vacuum under these conditions.

## 4 Discussion

### 4.1 Temperature and evaporation of the jet as it propagates in vacuum

As the jet expands into vacuum molecules will evaporate from the surface, cooling the liquid. To perform XUV spectroscopy it is essential that a vacuum is maintained, but also that there is knowledge of the temperature of the liquid as this may impact reaction rates and absorption features in large organic molecules. To this end we can calculate the cooling and evaporation from the sheet. The number of molecules evaporating from an area *A* is assumed to follow the Hertz-Knudsen equation:

$$\frac{\text{d}N}{\text{d}t}=\frac{A\left(p_{\text{vap}}-p\right)}{\sqrt{2\pi mk_{\text{b}}T}},$$ (2)

where *p*_vap_ is the vapour pressure of the liquid, *p* is the partial pressure of the vapour surrounding the liquid and *T* is the temperature of the liquid. Following the treatment in Heissler et al. (2014) [based on Faubel et al. (1988)], the change in jet temperature due to this evaporation is

$$\text{d}T=-\alpha \frac{H_{\text{vap}}}{C_{\text{p}}},$$ (3)

where *H*_vap_ is the enthalpy of vapourisation, *α* is the ratio of evaporated molecules to molecules remaining in the jet and *C*_p_ is the specific heat capacity of the liquid. Due to relatively high liquid flow rates in our jets, we assume the change in mass due to evaporation is negligible so we write *α* as:

$$\alpha =\frac{\text{d}N}{N_{\text{A}}M},$$ (4)

where *M* is the mass of the liquid in the jet. The change in temperature over time as the jet expands into the vacuum is therefore:

$$\frac{\text{d}T}{\text{d}t}=-˜\frac{H_{\text{vap}}A\left(p_{\text{vap}}-p\right)}{C_{\text{p}}N_{\text{A}}M\sqrt{2\pi mk_{\text{b}}T}}.$$ (5)

In order to model the distribution of vapour around the jet we start with the vapour density at position (*x*, *y*, *z*) due to a point source at the origin

$$\rho_{\text{vap}}=\frac{\rho_{\text{0}}}{x^{2}+y^{2}+z^{2}}.$$ (6)

We can then expand the point source into a rectangular surface of width *w* and height *h*, and find the vapour distribution at a distance *r* from the surface

$$\rho_{\text{vap}}\left(x,y,r\right)=\int_{-\frac{w}{2}-y}^{\frac{w}{2}-y}\int_{-\frac{h}{2}-x}^{\frac{h}{2}-x}\frac{\rho_{\text{0}}\left(x,y\right)}{x^{2}+y^{2}+r^{2}}.$$ (7)

**FIGURE 6:**
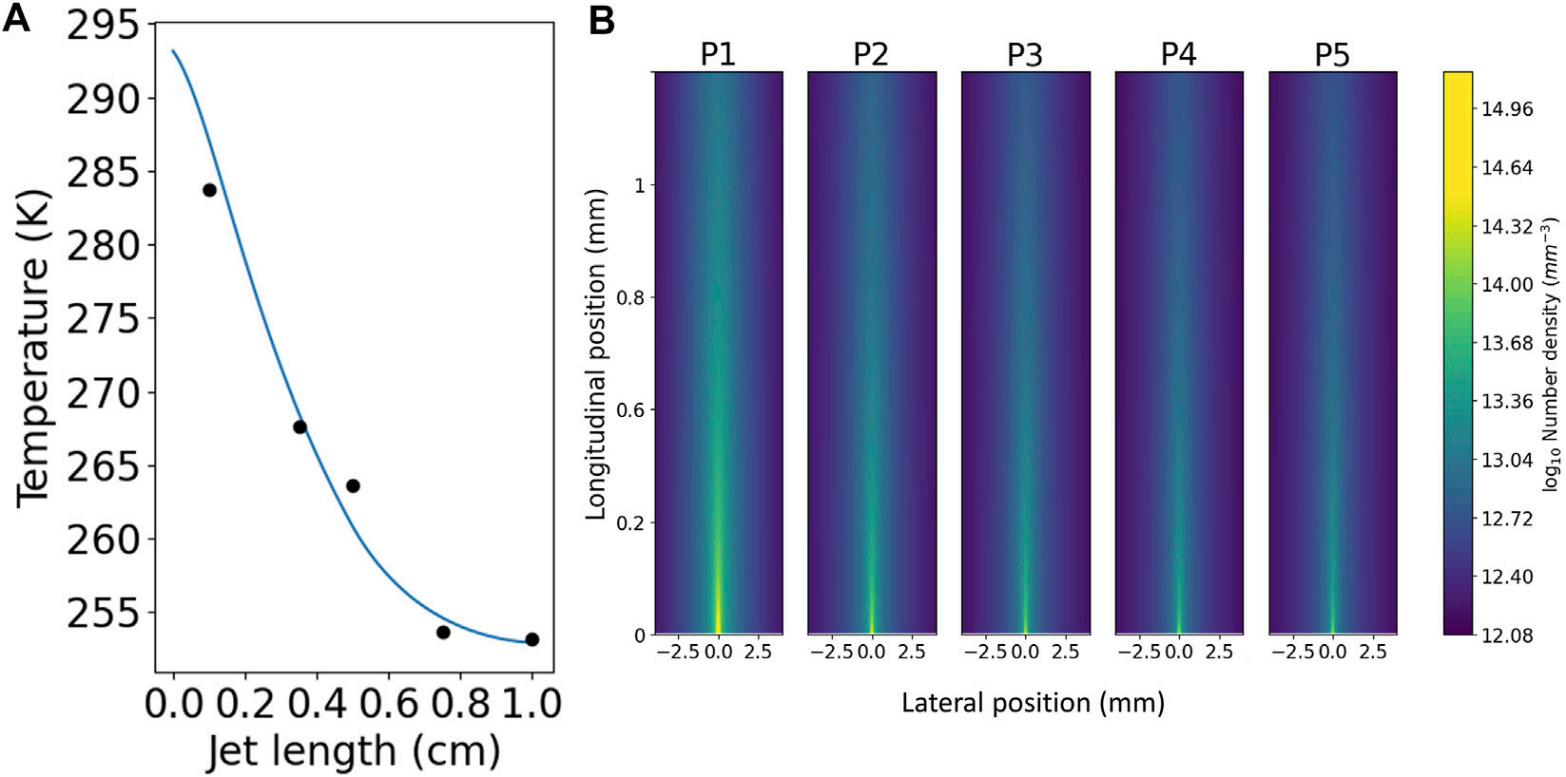
The relationship between jet length, temperature and pressure. **(A)** The blue curve shows the expected temperature decrease over it is length for a 1 cm long diamond shaped jet of isopropanol that is 1 mm wide at its middle with a flow rate of 9.1 ml/min. The black dots are experimental data taken under similar conditions, measured by manually inserting a thermocouple into the sheet. **(B)** The horizontal vapour distribution at the same vertical positions as the experimental data (listed as P1-5, with one being closest to the nozzle). As can be seen, by the bottom of the sheet (the thinnest and flattest part, ideal for taking measurements), the vapour density has dropped by two orders of magnitude.

Using these equations we calculate the temperature and consequent vapour pressure of the jet, as well as the distribution of the vapour, as can be seen in Figure 6. Recent work by the Wolf group (Chang et al., 2022) showed much more rapid evaporation and cooling in an alcohol than we have measured. This is probably due to the shape of the jet—theirs likely becomes much wider much more quickly, leading to a greater initial surface area and consequently faster evaporation and cooling.

### 4.2 Mitigation of freezing due to adiabatic expansion

**FIGURE 7:**
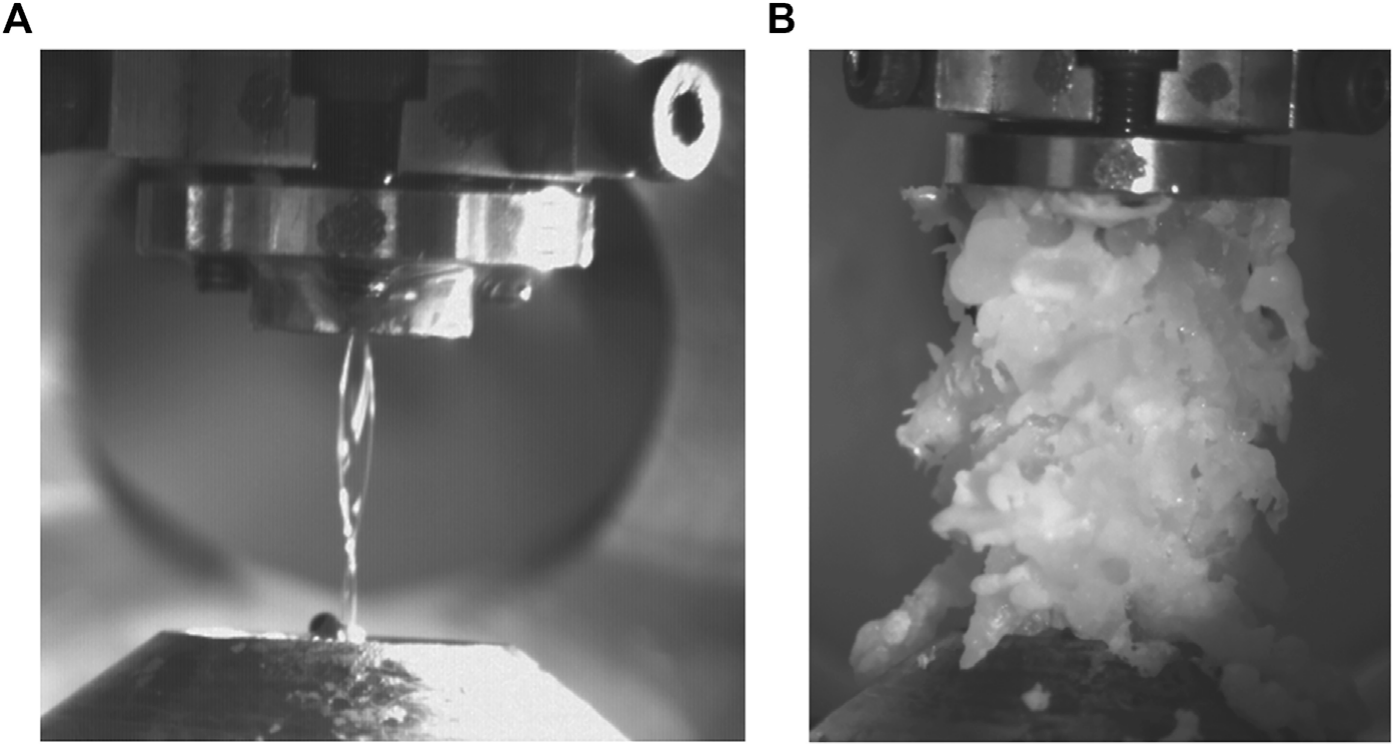
**(A)** before and **(B)** after images of catastrophic freezing in a jet of DMSO. The ice grows to this size in less than a second.

As the jet propagates in the vacuum it cools dramatically, cooling 40 K over a 1 cm distance (see Figure 6). If the temperature of the jet drops below the melting point then the liquid is at risk of freezing. If the critical point is at a pressure higher than the ambient pressure in the vacuum chamber, the material does not exist in the liquid phase at equilibrium. For example, we find water and dimethyl sulfoxide (DMSO) to be highly susceptible to freezing. Figure 7 shows unwanted freezing of DMSO which began at the catcher and quickly grew to cover the whole system. This not only halts the experiments but it can damage fragile nozzles, directly leading to failure in many cases.

In order to avoid the issue of freezing the collector tip can be heated—this is area where ice crystals are most likely to nucleate (see Figure 4B for details). Freezing can also be prevented by slowly decreasing the pressure in the chamber when going to vacuum. This avoids a rapid cooling of the sheet and vapour due to adiabatic expansion and has the advantage that alignment from nozzle to catcher is better preserved. This ensures the expansion process is as isothermal as possible, so the liquid is always evaporating rather than freezing, and avoids spillages of liquid into the chamber. It is important to do this with the jet running, as stationary water will freeze in the nozzle under vacuum.

## 5 Conclusion

Thin liquid sheet jets constitute a development of high utility for performing soft X-ray experiments in vacuum. Thin sheets allow the X-rays transmit with minimal reabsorption, and the opening of X-ray spectroscopy experiments to the liquid and solution phases allows a wider range of chemical and biological systems to be studied in their natural environment.

We have compared two types of thin liquid sheet jet, both of which make sheets thin enough for soft X-ray experiments. The fan spray nozzle will make sheets that are slightly thicker than the Micronit nozzle, but the sheets are also more stable with less structure due to surface waves. Both can be run in vacuum using the same apparatus, and if used with care can prove useful as targets for soft X-ray experiments in liquids. Nozzles represent a far lower setup cost than two colliding cylindrical microjets, the other widespread method of creating 1 µm thick sheets of liquid in vacuum, owing to the fact that they do not need to be aligned. The nozzle presented by Koralek et al. (2018) is commercially available, and therefore a good general solution, however for those with the resources to make the nozzle presented in Galinis et al. (2017), we recommend it as an alternative solution if larger target areas are desired.

## Data Availability

The raw data supporting the conclusion of this article will be made available by the authors, without undue reservation.
